# Impact of Library Preparation on Downstream Analysis and Interpretation of RNA-Seq Data: Comparison between Illumina PolyA and NuGEN Ovation Protocol

**DOI:** 10.1371/journal.pone.0071745

**Published:** 2013-08-19

**Authors:** Zhifu Sun, Yan W. Asmann, Asha Nair, Yuji Zhang, Liguo Wang, Krishna R. Kalari, Aditya V. Bhagwate, Tiffany R. Baker, Jennifer M. Carr, Jean-Pierre A. Kocher, Edith A. Perez, E. Aubrey Thompson

**Affiliations:** 1 Department of Health Sciences Research, Mayo Clinic College of Medicine, Rochester, Minnesota, United States of America; 2 Department of Cancer Biology, Mayo Clinic Comprehensive Cancer Center, Jacksonville, Florida, United States of America; 3 Department of Medicine, Mayo Clinic, Jacksonville, Florida, United States of America; University of Southern California, United States of America

## Abstract

**Objectives:**

The sequencing by the PolyA selection is the most common approach for library preparation. With limited amount or degraded RNA, alternative protocols such as the NuGEN have been developed. However, it is not yet clear how the different library preparations affect the downstream analyses of the broad applications of RNA sequencing.

**Methods and Materials:**

Eight human mammary epithelial cell (HMEC) lines with high quality RNA were sequenced by Illumina’s mRNA-Seq PolyA selection and NuGEN ENCORE library preparation. The following analyses and comparisons were conducted: 1) the numbers of genes captured by each protocol; 2) the impact of protocols on differentially expressed gene detection between biological replicates; 3) expressed single nucleotide variant (SNV) detection; 4) non-coding RNAs, particularly lincRNA detection; and 5) intragenic gene expression.

**Results:**

Sequences from the NuGEN protocol had lower (75%) alignment rate than the PolyA (over 90%). The NuGEN protocol detected fewer genes (12–20% less) with a significant portion of reads mapped to non-coding regions. A large number of genes were differentially detected between the two protocols. About 17–20% of the differentially expressed genes between biological replicates were commonly detected between the two protocols. Significantly higher numbers of SNVs (5–6 times) were detected in the NuGEN samples, which were largely from intragenic and intergenic regions. The NuGEN captured fewer exons (25% less) and had higher base level coverage variance. While 6.3% of reads were mapped to intragenic regions in the PolyA samples, the percentages were much higher (20–25%) for the NuGEN samples. The NuGEN protocol did not detect more known non-coding RNAs such as lincRNAs, but targeted small and “novel” lincRNAs.

**Conclusion:**

Different library preparations can have significant impacts on downstream analysis and interpretation of RNA-seq data. The NuGEN provides an alternative for limited or degraded RNA but it has limitations for some RNA-seq applications.

## Introduction

RNA expression profiling through next generation sequencing (RNA-seq) has become a standard approach in biomedical research due to its clear advantages over the traditional microarray based technology. Using the technology, many advances have been made in characterization and quantification of transcriptomes such as transcription start site mapping, strand-specific expression, fusion gene detection, expressed single nucleotide polymorphism/mutations, RNA editing, detection of alternative splicing events, and non-coding RNA identification [1]. RNA-seq is mostly carried out using polyadenylated (PolyA) tail selection, which uses oligo-dT affinity to select transcripts with the PolyA tail for sequencing. This step avoids high abundance RNA species that are not interesting to investigators such as rRNA, tRNA or histone mRNAs. However, this protocol requires high quality (fresh or fast frozen tissue) and sufficient amount of RNA from a sample to be sequenced, which is very challenging for partially degraded RNA from the rich archive of formalin fixed paraffin embedded (FFPE) samples and a tiny tissue from biopsy. Additionally, evidence shows that some interesting RNAs do not have the PolyA tail [2] and the PolyA selection would be inappropriate in such case. To address these limitations, several new library preparation protocols have been developed for RNA-seq, among which the NuGEN (San Carlos, CA, USA) Ovation RNA-Seq System is commonly used. This protocol can simplify library preparation and uses as little as 500 pg of total RNA, either from limited RNA source or partially degraded RNA from FFPE. The protocol uses a single primer isothermal amplification to amplify RNA target into cDNA before standard Illumina library preparation that includes end repair, A-tailing, and ligation of selected sequencing adapters [3], [4], [5], [6]. For example, Wu and colleagues successfully applied WT-Ovation Pico RNA Amplification System to deep-sea microbial samples with very low cell density and high impurity for metatranscriptome analysis. Using pooled bronchial airway epithelial cell brushings, Beane et al. conducted RNA-seq for the libraries prepared by both the Illumina PolyA selection and the NuGEN Ovation System and demonstrated fairly good agreement for the common genes detected by both libraries (Pearson correlation r 0.59) [6]. In spite of these observations, questions remains considering the broad applications of a RNA-seq experiment: 1) how do the library preparations affect differentially expressed gene detection across biological conditions, one of the most important and common questions from the RNA-seq; 2) do the library preparation affect expressed single nucleotide variant (SNV) detection? 3) Does the NuGEN library provide advantages over the PolyA in non-coding RNA detection?

With those questions, we conducted an RNA-seq experiment for 8 breast cell lines whose sequence libraries were prepared by both the Illumina PolyA selection and the NuGEN Ovation RNA-Seq kit. We compared their respective alignment efficiencies, gene detection/quantification, differentially expressed gene detection between biological replicates, and SNVs. Although the results from the two protocols largely agreed, clear differences were observed. In-depth analyses were performed to elucidate the differences and recommendations were provided for library preparation protocol selection, data analysis and interpretation.

## Materials and Methods

### Samples and RNA Preparation

RNA from eight human mammary epithelial cell (HMEC) lines obtained from American Type Culture Collection (ATCC) was extracted from mid log phase cultures at low passage (P1 or P2) using TRIzol kit (Invitrogen), each with high quality RNA (RIN numbers all >9.7). These cell lines were derived from 8 different individuals (biological replicates), of which 4 were with involuted and 4 were with non-involuted epithelial cells, the phenotype associated with an increased breast cancer risk [14]. For NuGEN libraries, 500–800 pg total RNA were amplified and converted to cDNA using NuGEN’s Ovation RNA-Seq kit. Following amplification, 4.6–5.0 ug cDNA was fragmented to ∼200 bps using Covaris S2 and the fragmentation parameters described in the NuGEN ENCORE NGS library preparation protocol. The remainder of the library preparation followed manufacturer’s protocol as described in NGS Library System I and Multiplex System, Part no. 300. For Illumina libraries, 5 ug total RNA were processed according to the mRNA Seq Sample Preparation Kit protocol (part number 1004898 Rev A), with the 300 bp gel fragment eluted and purified for sequencing, as described in Sun et al [7].

### RNA Sequencing

The paired-end sequencing at 51 cycles (50 bases each end) was carried out for both the NuGEN and Illumina PolyA preparations. The former was conducted by GAIIx sequencer and the later was by HiSeq 2000 at Mayo Clinical Medical Genomics Facility. The raw sequence data has been deposited to GEO with accession number GSE47933.

### Sequence Alignment and Gene Level Expression Quantification

Sequence alignment and quantification of gene and exon level expression was carried out using internally developed RNA-seq analytical pipeline. Briefly, the pair end reads were aligned to the human genome build 37.1 using TopHat (1.4.0) and Bowtie (0.12.7). HTSeq (0.5.3p3) was used to perform gene counting while BEDTools (2.7.1) was used to count the reads mapping to individual exons according to RefSeq gene annotations (Feb 2009, GRCh37/hg19) with 23,113 genes. Gene level expression was normalized by reads per kilobase per million mapped reads (RPKM) [8] for filtering genes and comparing correlation between two library preparations.

### Differentially Detected/Expressed Gene Detection

We compared both differentially detected genes between two library preparations (8 vs. 8 samples of two library preparations), and differentially expressed genes between two biological conditions (4 vs. 4 samples of two different phenotypes) within the same library preparation and then compared the commonly detected or uniquely detected by either platform. For both tests, we applied count based negative binomial model implemented in the R package “edgeR” [9], in which normalization factor was calculated by trimmed mean of M values (TMM) method [10]. This normalization constant was incorporated into the models to account for varying library sizes. The gene-wise dispersions were estimated by conditional maximum likelihood and an empirical Bayes procedure was used to shrink the dispersions towards a consensus value. The differential expression was assessed using an exact test adapted for over-dispersed data for the cell line samples with two different phenotypes. For the comparison between samples prepared by PolyA and NuGEN (differentially detected genes), we treated them as paired and applied Cox-Reid profile-adjusted likelihood method in estimating dispersions and likelihood ratio test for differentially detected genes.

### Single Nucleotide Variant Calls

One of the major advantages in RNA-seq data is to detect expressed single nucleotide variants (SNVs) from the sequence reads. However, it is not clear how different library preparations may affect these discoveries. To answer this question, we applied SNVMix2 [11], a probabilistic Binomial mixture model to infer SNVs from NGS data and GATK UnifiedGenotyper [12] for SNV calling. For SNVMix2, we used the minimum coverage of 4 and the combined heterozygous and homozygous alternative allele probability (AB+BB) >0.8 for a variant allele. The default settings were used for GATK. We compared the number and overlap of the SNVs called in each sample between the PolyA and NuGEN preparation and between variants detected by different callers. The variants were further categorized according to their genomic locations and functional impacts.

### Intronic Gene Expression

As high proportion of reads was mapped to the genomic regions beyond RefSeq exon annotations in NuGEN samples, we also evaluated the “expression” of intragenic regions (introns) for each RefSeq gene with two or more exons. To do this, the hg19 refFlat file downloaded from UCSC was used to create a intragenic segment GTF file. This file was then passed to HTSeq (0.5.3p3) to count the total number of reads mapped to introns of a gene using the same aligned bam files as the gene expression described above. Total and proportion (of library size) of reads mapped to these regions, the number of genes with intragenic expression, and variance of intragenic expression were compared between PolyA and NuGEN samples.

### Expanded Gene Quantification with GENCODE Annotations

hg19 RefSeq annotation has 23,113 well annotated genes. However, many less characterized or novel genes, particularly non-coding RNAs, are not included in the annotations. To test whether there is an edge to capture these additional genomic features by the NuGEN protocol, we expanded our analyses to GENCODE annotations (v12, Ensembl 67) with 11,790 long noncoding RNAs, 8801 small RNAs, and 12, 869 pseudogenes by replacing RefSeq annotation with this combined GENCODE/Ensembl annotation in gene expression calculation with HTSeq. The number of genes in each gene functional category was compared between the PolyA and NuGEN samples.

### Novel lincRNA Prediction from the Two Protocols

In addition to the annotated lincRNAs in GENOCODE, we were also interested in potential novel lincRNAs detected from each protocol. To do this, we applied our lincRNA analytical pipeline to focus on new lincRNAs not defined in GENOCODE. Briefly, after the Tophat alignment, we used Cufflinks and Scripture to name the common candidate transcripts (the commands and parameters were provided in the Table S2). For a transcript to be a potential lincRNA, it needed: 1) at least 200 bp long; 2) a transcript with at least 3 supporting reads 3) no overlap with protein coding region/domain; 4) no protein coding potential as predicted by CPAT [13] and no blastp hits. Only the lincRNAs that are not present in the GENCODE annotations were considered for this comparison. Novel lincRNAs in each sample were compared with other samples and lincRNAs with the same or similar coordinates were merged and then used for quantification of lincRNA expression.

### RT-PCR Quantification for Selected Genes

We conducted RT-PCR for 5 genes (*AR, ESR1, HGF, SIX1, and TWIST1*) that were implicated in breast epithelium involution using GAPDH as an internal control. RNA extraction was performed with the Qiagen mRNeasy kit, including on-column treatment with DNase; and cDNA conversion was carried out using the Applied Biosystems cDNA Archive protocol, according to the manufacturers’ recommendations. Reverse transcriptase-mediated quantitative real time PCR (qPCR) was carried out using commercially available primer/probe/target reagents obtained from Applied Biosystems (ESR1 Hs01046818_m1, AR Hs00907244_m1, GAPDH Hs99999905_m1, HGF Hs00300159_m1, SIX1 Hs00195590_m1, TWIST1 Hs00361186_m1). Real time amplification kinetics were collected using the Applied Biosystems HT7900 cycler and data are represented as ΔCT using GAPDH as a reference control.

## Results

### Mapping Efficiency, Biases and Number of Captured Genes

**Figure 1:**
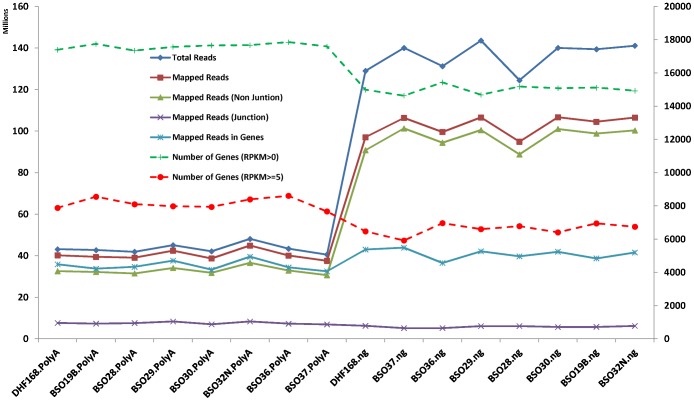
Alignment and Mapping Statistics for 8 HMEC. Y axis on the left for solid lines and Y axis on the right for dashed lines, representing millions of reads and numbers of genes captured, respectively. The samples from PolyA preparation are on the left side of the graph (with “.PolyA” extension on sample name) and the NuGEN samples are on the right (with “.ng” extension). Although the NuGEN samples had much higher sequence depth, the numbers of reads mapped to exon/gene were quite similar to the PolyA samples or slightly. The numbers of captured genes were lower in the NuGEN samples.

For the 8 HMEC cell line samples, we used the same RNA extracts for both NuGEN and Illumina PolyA library preparation. The PolyA library was sequenced with the Illumina GAIIx sequencer and about 40 millions of pair-end reads (2×50) were generated each sample. The NuGEN library was sequenced with the Illumina HiSeq 2000 where each sample had 120–140 millions of paired reads. Both data were in good quality as assessed by FastQC (http://www.bioinformatics.babraham.ac.uk/projects/fastqc/). The base quality from position 1 to 50 was quite uniform with median, mean, and lower 25 percentile all >30 (Figure S1). The mapping efficiencies for the PolyA library ranged from 91.8% to 94.2% while the efficiency was lower for the NuGEN library (74.2% to 76.3%). In spite of higher numbers of reads mapped in the NuGEN samples than in the PolyA samples, the numbers of reads mapped to the annotated genes including exons and junctions (according to RefSeq gene annotations) were only slightly higher in the NuGEN than in the PolyA samples ((36.5–43.8 million vs. 32.5–39.9 million vs., Figure 1), which account for ∼40% and 90% of the aligned reads, respectively. While 18–20% of reads were aligned to exon-exon junctions in the PolyA samples, only 5–6% of reads in the NuGEN samples were in exon-exon junctions. The reads mapped to rRNAs and tRNAs were slightly higher in the NuGEN samples than the PolyA samples (0.57–1.21% and 0.2–0.4% vs. 0.52–1.1% and 0.004–0.012%, respectively).The estimated average insert sizes (the inner distance between the two read pair) were 100 and 0.25 for the PolyA and the NuGEN samples, respectively. The number of genes detected in each sample in the NuGEN samples run from 14,628 to 15,429 while this number was much higher in the PolyA samples, ranging from 17,341 to 17,850 (12.4∼21.3% more in the PolyA samples) when standardized RPKM (reads per kilobase per million mapped reads) [8] expression greater than 0 was used. With RPKM> = 5, the similar pattern was observed (5,912∼6958 vs. 7,660∼8,602, 13.7∼44.6% more, Figure 1, y axis on the right). These data showed that sequence reads from the NuGEN protocol had lower mapping rate, lower fraction of reads mapped to coding regions and exon-exon junctions, and lower number of genes detected.

### Impact of Library Preparation on RNA Quantification

**Figure 2:**
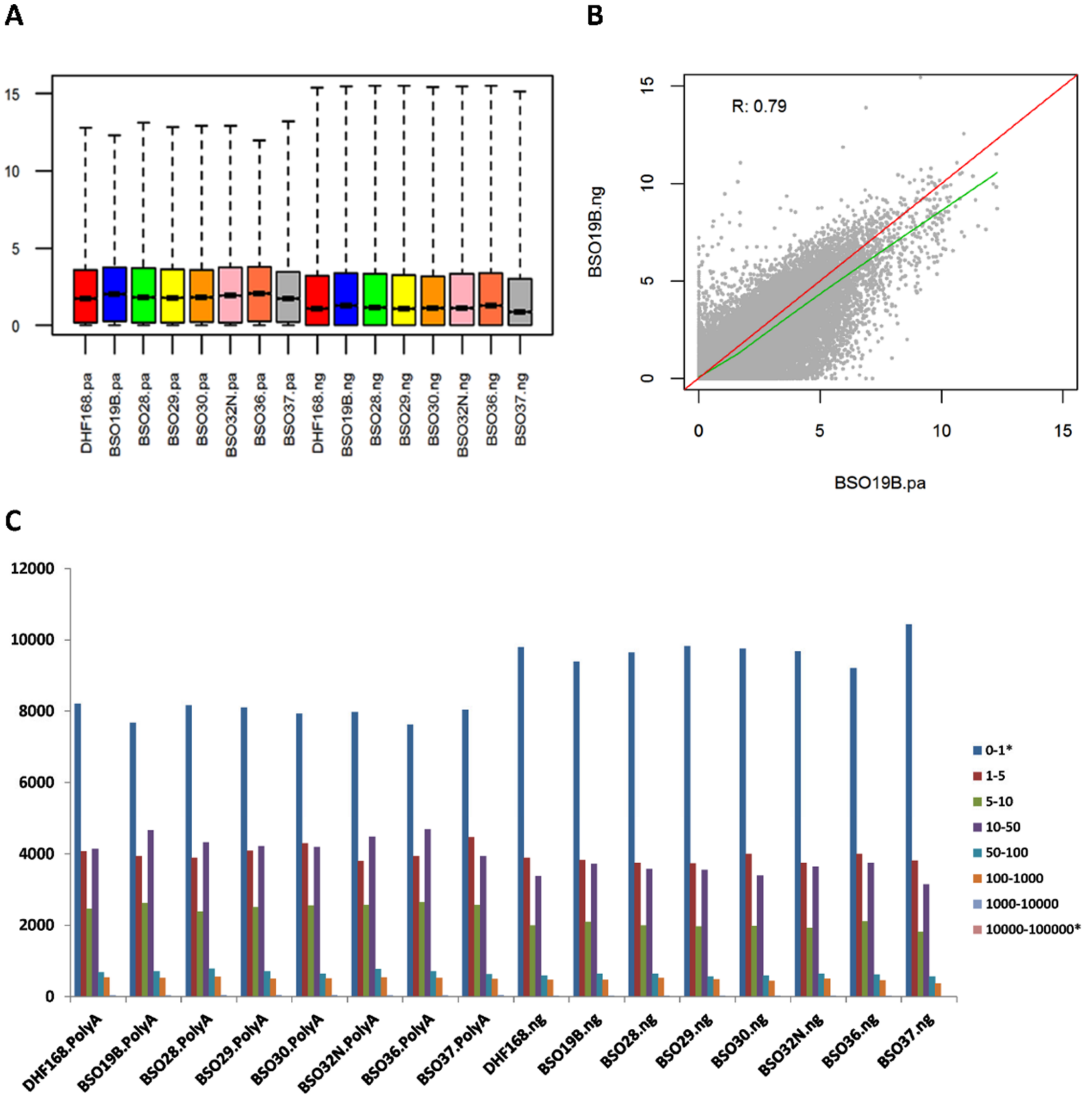
Gene expression distribution, correlation, and detected genes. A: Boxplot of RPKM normalized expression for 8 samples from the PolyA and 8 samples from the NuGEN library preparation (from left to right in the same order). Samples with the same color were the same RNA extract but prepared by either the PolyA or NuGEN protocol. B: The example scatter plot of RPKM expression of the same sample (sample BSO19B) between two library preparations. R - Pearson correlation coefficient. Red line – diagonal line for perfect correlation. Green line – fitted lowess cure for the data. C: Gene counts in different expression level binned by standardized expression RPKM. There were 20,156 genes detected in at least one sample and all these genes were used for the binning. Y-axis: number of genes detected in each expression range in RPKM. Samples on the left side are the PolyA and on the right are the NuGEN. NuGEN samples had more genes missed (in 0–1 range) than PolyA samples. Two genes are highly expressed (>10,000 rpkm) in the NuGEN samples but no genes were in this range in the PolyA samples. The ranges with * had more genes in the NuGEN samples than the PolyA samples.

Because the two library preparations were sequenced at different depths, we first normalized the gene level expression data using RPKM (1 million of reads mapped to genes, not aligned reads) to assess the agreement between the two libraries in terms of number of genes captured and measurement of gene expression level. Since the two library preparations used the exact RNA extract for each sample and the difference between the two would be mainly due to technical deviations. Among the 23,113 RefSeq genes, 2,957 did not have any reads mapped in any of the 16 samples (8 PolyA and 8 NuGEN samples) and they were first removed, which led to a total of 20,156 genes for further assessment. Figure 2A is the boxplot of log2 RPKM (after adding 1 to all genes before log2 transformation) for the 16 samples. As clearly seen, the gene expression in the samples from the NuGEN library had a wider range of expression: the lower 25% (the first quarter) of genes had no expression (at 0 line) while a few genes were expressed at very high level. The correlation of log2 RPKM gene expression between the paired PolyA and NuGEN samples was moderately high (Pearson correlation r from 0.71 to 0.81 and r^2^ from 0.5 to 0.65, an example sample BSO10B was shown in Figure 2B). There was an overall trend that the detected gene expression was higher from the PolyA protocol than the NuGEN (the fitted lowess curve tilted to x-axis). Through binning genes into 8 levels of expression at RPKM of 0–1,1–5, 5–10, 10–50, 50–100, 100–1000, 1000–10,000, and 10,000–100,000 (exclusive for lower bound and inclusive for upper bound except 0–1 where 0 was included), we counted the numbers of genes in each bin and found that except for the lowest and the highest bins where the NuGEN samples had more genes, all other bins had more genes in the samples from the PolyA samples (Figure 2C). There were 2,160 genes that were not detected in any of the NuGEN samples but in the PolyA samples; conversely there were 376 genes not detected in any of the PolyA samples but only in the NuGEN samples. The similar statistics were observed for the raw count data without RPKM normalization. These results showed that the genes captured by the PolyA preparation were more diverse while the NuGEN library preparation missed a significant number of genes at different levels of expression.

### Differentially Measured Genes between the Two Platforms

The PolyA and the NuGEN library preparations are different protocols and it is expected each may work well for some genes but not others. It is important to know how these genes are differentially measured. To this end, we compared the differentially detected genes between the replicates from the two different library preparations using generalized linear model for count data implemented in edgeR [9], treating the same sample from the two libraries as a pair. In this case the raw count data, not RPKM data, were used. The different library sizes were normalized internally using the TMM method (weighted trimmed mean of M-values), which calculates a scaling factor for each sample to a reference sample whose upper quartile is the closest to the mean upper quartile of the samples after removing outliers. The TMM minimizes the impact of extreme values and performs better than library size or upper quartile normalizations [10]. At false discovery rate (FDR) <0.05, 12,650 (out of 20156) or 63% genes were differentially measured between the two platforms, among which 8,523 (42%) have fold change >4. About twice more genes were detected higher in the PolyA samples than in the NuGEN (8,298 vs. 4,352) samples and the differences were mainly in the low to intermediately expressed genes, many of which were only captured in the samples from one library preparation but not another.

### Impact of Library Protocols on Differentially Expressed Genes (DEGs) between Biological Conditions

**Figure 3:**
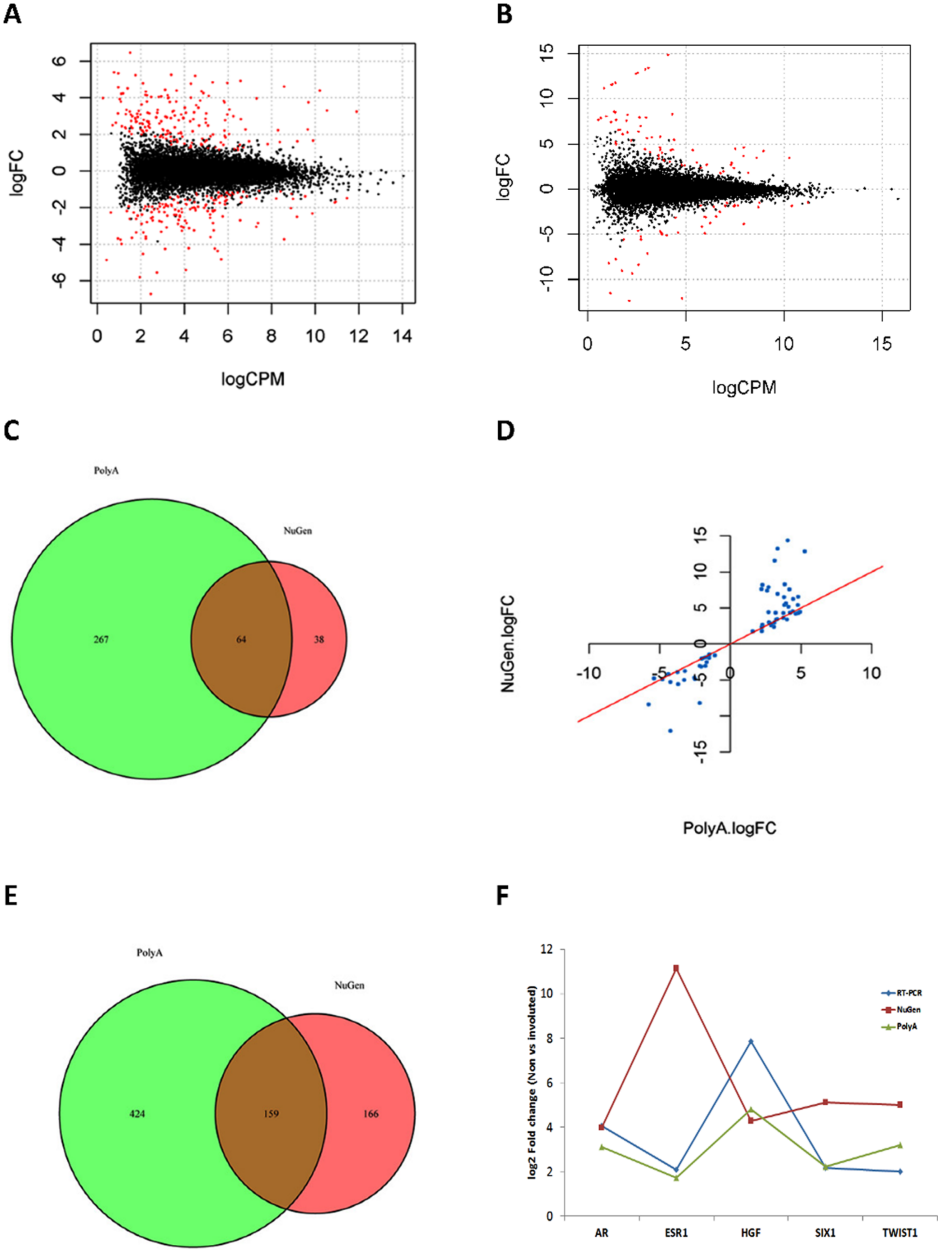
Differentially expressed genes in biologic replicates. A: Differentially expressed genes at FDR 0.05 (highlighted in red) between non-involuted and involuted epithelial phenotypes of the PolyA samples by edgeR. X-axis is the average expression across groups (normalized at count per million) in log2 scale. The Y-axis is the log2 fold change between non-involuted and involuted samples. B: Differentially expressed genes at FDR 0.05 (highlighted in red) between non-involuted and involuted epithelial phenotypes of the NuGEN samples by edgeR. X-axis is the average expression across groups (normalized at count per million) in log2 scale. The Y-axis is the log2 fold change between non-involuted and involuted samples. C: Venn diagram for common and unique DEGs (differentially expressed genes) by the PolyA and the NuGEN preparations at FDR 0.05. D: The scatter plot of log2 fold changes for the 64 common genes in 3C. All genes are in the same direction and most genes agree very well in fold change estimate except some genes have higher fold changes in the NuGEN samples. E: Venn diagram for common and unique DEGs (differentially expressed genes) by the PolyA and the NuGEN preparations at raw p value <0.01. More genes are significant and overlapping but the common genes are in the similar proportion as with FDR cut-off (21% vs 17% of total DEGs by either). F: Comparison of 5 genes between RT-PCR, DEGs by the PolyA preparation, and DEGs by the NuGEN preparations. All genes are differentially expressed by three methods using un-adjusted p value. The fold change estimates agree better between the PolyA preparation and the RT-PCR than between the NuGEN preparation and RT-PCR.

One of the critical questions in different library preparations is to what extent they will affect DEGs between biological conditions if one preparation method is used versus another. In the 8 HEMC samples, 4 samples were derived from involuted and other 4 were from non-involuted epithelial cells, the phenotype associated with an increased breast cancer risk [14]. We were interested in the DEGs between the two phenotypes and what would be the impact from the library preparations for the detection. For these comparisons, we first filtered out the genes at very low expression (reads per million < = 4 in 6 or more samples) in each library preparation (12,244 and 11,321 genes passed the filter for the PolyA and the NuGEN samples, respectively) and then conducted DGE detection separately for each library data between the two cell line phenotypes using edgeR [9]. At false discovery rate (FDR) cutoff of 0.05, there were 331 genes that were differentially expressed by the PolyA protocol (Figure 3A) while 102 genes were significant at the same criteria by the NuGEN protocol (Figure 3B). The common significant genes from the two library preparations were 64, which accounts for 19% and 63% of significant genes claimed in the PolyA and the NuGEN samples, respectively (Figure 3C). In spite of the low overlap with the PolyA samples, the estimated fold change and directions were highly comparable for these commonly changed genes (Figure 3D). When the raw p value less than 0.01 (not affected by the number of genes in testing) was used as the cut-off, the PolyA samples had 583 significantly changed genes while the NuGEN samples had 325 significant genes with 159 overlapping genes between the two (27% of the PolyA and 49% of the NuGEN, Figure 3E). Similar results were obtained when all genes without any filtering or the commonly detected genes (10,388) in both protocol preparations were used (data not shown). Among the DEGs, we had 5 genes (*AR, ESR1, HGF, SIX1, and TWIST1*) with RT-PCR data for validation. Four of the five genes were differentially expressed from the RT-PCR experiment at p value <0.05 (Student’s T test for ΔΔCTs) and one (*ESR1*) was borderline (p = 0.07). All the five genes were differentially expressed in both the PolyA and the NuGEN samples; however, the estimated fold changes in the PolyA samples were closer to RT-PCR data than the NuGEN samples (Figure 3F).

### Impact of Library Preparations on SNV Detection

**Figure 4:**
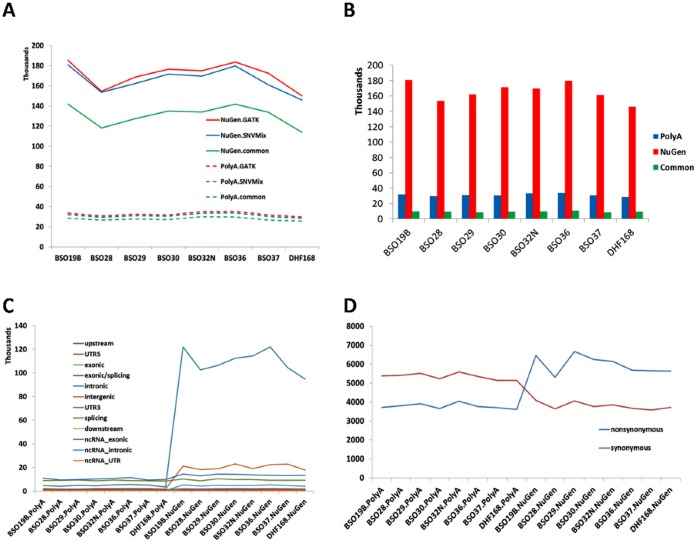
SNV detection comparison between the two library preparations. A: The total number of SNVs by the NuGEN (solid lines) and the PolyA (dashed lines) preparation (all SNVs regardless of genomic positions). There are dramatic differences between the two library preparations. However, the differences between two callers, SNVmix and UnifiedGenotyper, are small. X-axis –samples. Y-axis – number of SNVs detected in millions. B. Detected SNV comparison between the PolyA and the NuGEN preparations. The common SNVs are low. C. The number of SNVs by genomic positions for each sample by the PolyA (left) and the NuGEN (right). Higher numbers of SNVs were detected in the NuGEN samples than the PolyA samples, particularly in the intragenic and intergenic regions. D. SNVs in the coding regions by functional impacts in the PolyA and NuGEN samples. Although the total SNVs in the coding region are similar, SNVs from the PolyA have more synonymous SNVs than non-synonymous SNVs yet these are reversed for the NuGEN samples.

The detection of SNVs from RNA-seq data has an exceptional value that allows to identify expressed variants or mutations from sequenced samples such as tumors [15], [16] or explore RNA editing events (or RNA DNA difference). However, it is not clear how different library preparations may affect these discoveries. To answer this question, we applied SNVMix2 [11], a probabilistic binomial mixture model to infer SNVs from NGS data and GATK UnifiedGenotyper [12], for each sample for SNVs. The variants detected in the PolyA samples were around 30,000 per sample (28,491–33,741 for SNVmix2 and 29,778–35,190 for GATK), among which 85–90% were called by both. However, there were 5–6 times more SNVs (146,001–180,827 for SNVmix2 and 149,840–185,371 for GATK) detected in the NuGEN samples (Figure 4A, B). The common SNVs detected by both algorithms ranged from 75–83%. However, when comparing the SNVs in the same samples between the two libraries, about 30% (27.4–32.3%) of SNV positions detected in the PolyA samples were found in the NuGEN samples, which only accounts for 5% (5.1–6.3%) of total SNVs in the latter samples. For the SNV sites captured by both library preparations, over 99.6% were in concordance. When we classified the SNVs according to their genomic locations, the numbers of SNVs in the coding regions were very similar between the PolyA and the NuGEN samples; however, the SNVs in non-coding regions were much higher in the NuGEN samples, particularly in the intragenic and intergenic regions where there were 23.5 and 12.7 fold more SNVs, respectively (Figure 4C). In the coding regions, more synonymous SNVs were observed in the PolyA samples while more non-synonymous SNVs were seen in the NuGEN samples (Figure 4D). About a third (26–31%) of coding region SNVs were commonly detected by both the PolyA and the NuGEN library preparation. For the SNVs detected in the PolyA but not in the NuGEN, majority of them were due to low or no coverage in the NuGEN samples. For variants detected in the NuGEN but not in the PolyA, there were several reasons we observed: 1) there were no sequence reads in intragenic or intergenic regions in the PolyA samples yet many reads were generated in the NuGEN samples. 2) No alternative allele was seen in the PolyA but only in the NuGEN sample.

### Exon Capture, Base Level Coverage and 3′ Coverage Bias between the PolyA and the NuGEN Preparations

**Figure 5:**
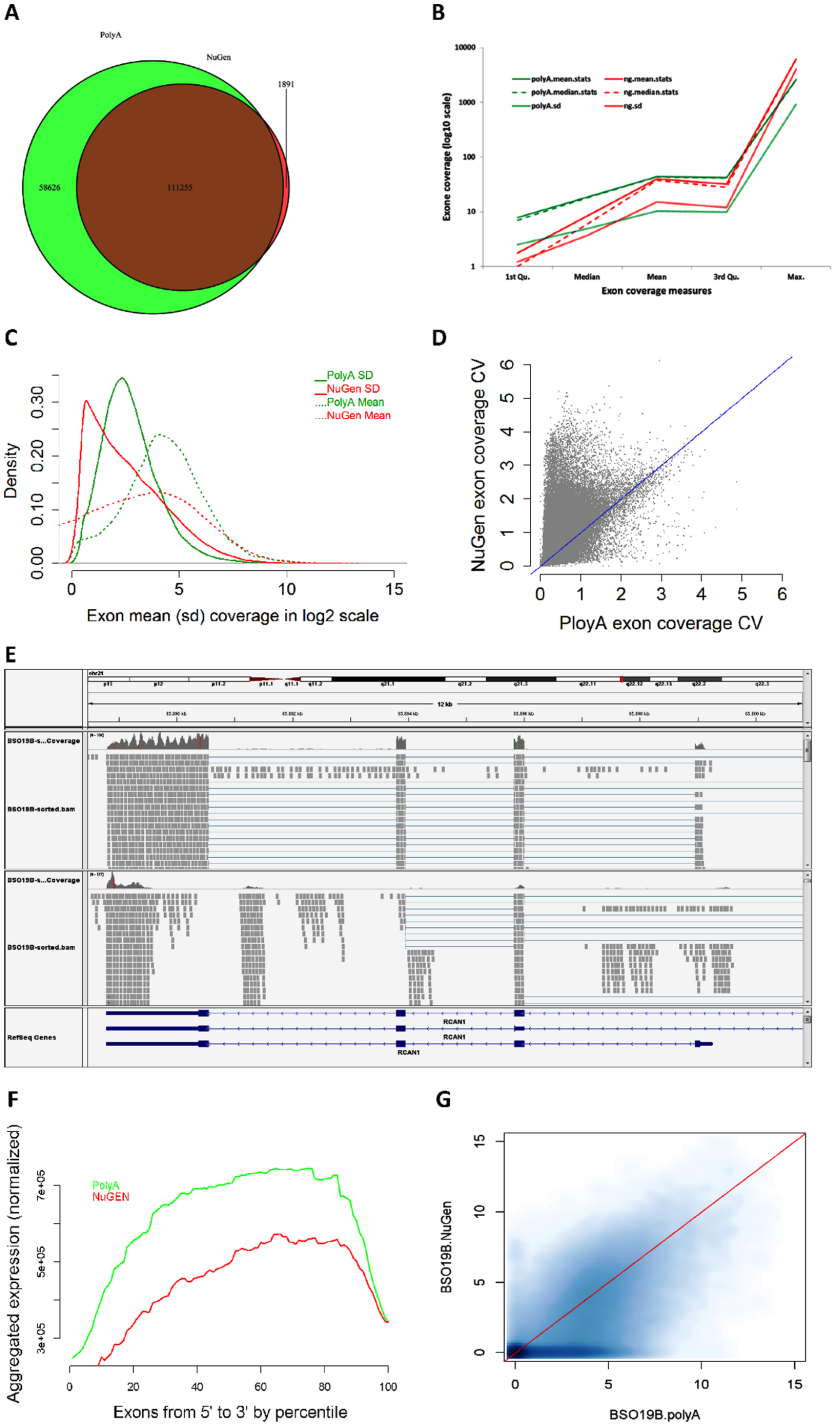
Exon and exon base coverage between the PolyA and the NuGEN preparations. A: Captured exons by either the PolyA or the NuGEN with the common and unique ones shown in the Venn diagram. B: For commonly captured exons (111,255), the PolyA have higher and more even coverage than the NuGEN sample. C: Compared to the PolyA library, the average exon coverage for the NuGEN is more spread, with many exons at very low coverage (higher density on the left) while a few with very high coverage (long tail on the right side). Although the low variance at the low covered exons, the variance for highly covered exons are larger than the PolyA library. D: The coefficient of variance in majority of exons is higher in the NuGEN preparation than the PolyA preparation. Blue line – diagonal line. E: An example exon coverage for gene RCAN1. The upper panel is from the PolyA library preparation and the lower is from the NuGEN preparation. Uneven coverage is more obvious in the NuGEN than in the PolyA. A exon skip in the NuGEN sample is also seen. Noted also is there are quite a few reads mapped into the intronic regions in the NuGEN sample. F: 3′ bias at transcript level, both in the PolyA and the NuGEN preparation. The NuGEN appears more obvious. The data is normalized by RPKM at exon level and the transcript level is standardized at percentile. G: The aggregated expression (coverage) from the NuGEN is lower than the PolyA.

To further elucidate the differences between the two library preparations, we then compared the exon level coverage using one of the samples as an example (BSO19B). There are a total of 224,165 exons in RefSeq annotations for hg19. For the PolyA sample there were 169,881 exons (75.8% of total) with at least 1 read covered whereas the NuGEN sample only had 113,146 (50.5%), 25% less than the PolyA sample. The commonly covered exons were 111,255, which leads to 58,626 exons missed by the NuGEN preparation and 1,891 exons missed by the PolyA preparation (Figure 5A). For each individual exon covered by both the PolyA and the NuGEN, we obtained its base level coverage statistics for each exon such as mean, median, 1^st^ and 3^rd^ quartile, and max coverage. As shown in Figure 5B, the exon coverage from the PolyA (green lines) had higher median, median, 1^st^ and 3^rd^ quartile except the maximum coverage than the NuGEN sample (red lines). Noted also is that the variance of coverage (shown as standard deviation in Figure 5B) for exons were generally lower in the PolyA sample than the NuGEN sample, an indication of more even coverage across different positions in an exon in the PolyA sample than the NuGEN sample. The exon average coverage for the NuGEN sample is more spread, with many exons at very low coverage (high density around 0, Figure 5C) while a few with very high coverage (long tail on the right side). Although the low variance at the low covered exons, the variance for highly covered exons were larger in the NuGEN sample than the PolyA sample. The coefficient of variance in majority of exons was higher in the NuGEN sample than the PolyA sample (Figure 5D). These results suggest more uneven coverage in the NuGEN than in the PolyA sample in addition to exon skip. An example of such patterns was visualized in the IGV (Integrative Genomics Viewer) [17] for gene *RCAN1* (Figure 5E). Additionally, we also evaluated the overall transcript level coverage from 5′ to 3′ end for potential bias from the protocols (not as the result of RNA degradation) by standardizing exon coverage using RPKM and a different number of exons in different genes by percentile. As shown in Figure 5F, both the PolyA and the NuGEN had a clear 3′ bias and the coverage at the gene body for the PolyA sample appeared more even than the NuGEN sample. The aggregated coverage from all genes in the NuGEN sample was lower than the PolyA sample as many exons were not captured or had a lower coverage (Figure 5G, the darker cloud from 0 to 5 on x-axis below the diagonal line).

### Intragenic Gene Expression

**Figure 6:**
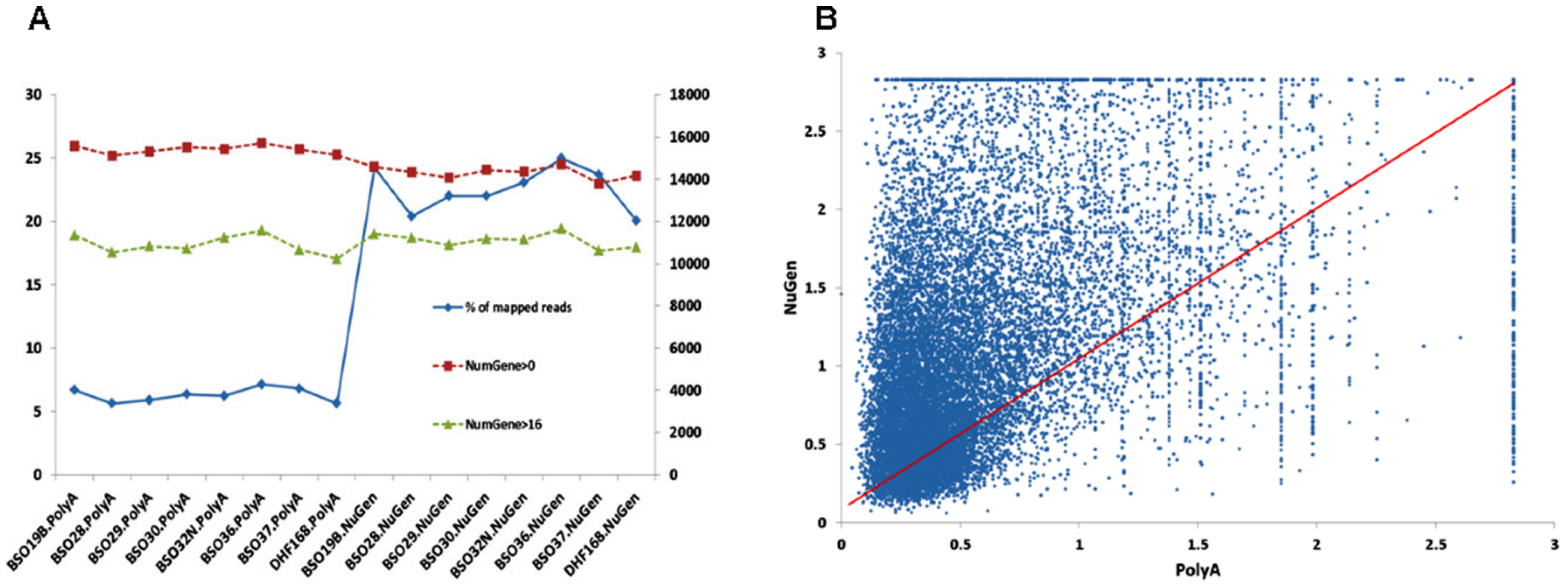
Intragenic expression of the PolyA and the NuGEN samples. A: The percentage of mapped reads to intragenic regions are much higher in the NuGEN than the PolyA samples (left axis for the blue line), however the number of genes with detected intragenic expression is lower than or similar to the PolyA samples. B: intragenic expression coefficient of variance between the PolyA and the NuGEN samples. The NuGEN samples have much higher variance than the PolyA samples.

As observed above, although significant higher numbers of sequence reads were mapped to the genome in all the NuGEN samples, the numbers of reads mapped to annotated RefSeq genes were similar to the PolyA samples (Figure 1). One of the possible reasons was many of these reads might have been mapped to intragenic regions. We tested this hypothesis by evaluating the intragenic “expression” for the same set of RefSeq genes. For the PolyA samples, about 2.5 million of reads (ranging from 2.2 to 2.8 million) or 6.3% of total mapped reads were in the intragenic regions. However, 20–25% of total reads (19.5–25.3 million) were mapped into the intragenic regions for the NuGEN samples. In spite of the significant higher numbers of reads in the intragenic regions, the numbers of genes with intragenic expression in the NuGEN samples were still lower or similar to the PolyA samples (Figure 6A). Similarly, the intragenic expression among the 8 NuGEN samples was highly variable compared to the PolyA samples as measured by the coefficient of variance among the samples in the same library preparation (Figure 6B).

### Impact of Library Preparations for “Novel” Gene Detection

**Figure 7:**
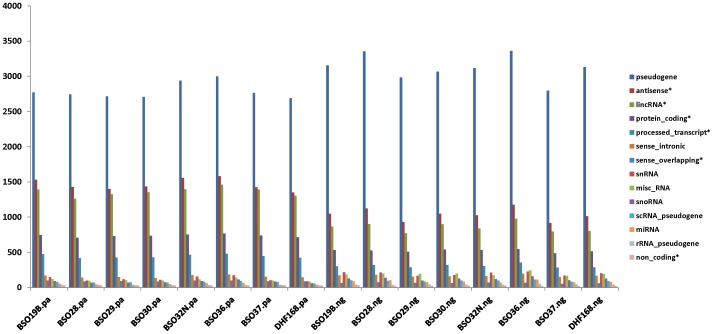
Additional genes detected in each sample by gene categories. Samples with “.pa” extension were from the PolyA preparation and the samples with “.ng” were from the NuGEN preparation. Y-axis – number of genes with at least 1 read mapped. Gene categories with * in the legend have higher numbers in the PolyA samples and those without are higher in the NuGEN samples.

One of advantages of the NuGEN protocol is potentially to identify genes that are not PolyA tailed. To test this hypothesis, we mapped reads to the expanded gene annotations from the latest GENCODE project, which has more than doubled number of genes (52,401) including thousands of lincRNAs (5,690) and miRNAs (1,586) compared to the RefSeq gene annotations (common genes between: 20,073). We first excluded the common genes with the RefSeq and then compared the additional genes detected by either preparation according to the gene categories. There were about 7,600 additional genes in the PolyA samples with at least one read mapped in any of the 8 samples versus 6,900 additional genes in the NuGEN samples. The number of detected genes was higher in most of the RNA categories such as protein coding, lincRNAs, antisense RNA (Fig. 7 with *) in the PolyA samples than in the NuGEN samples. On the other hand, the NuGEN preparation appeared picking slightly more other types of genes such as pseudogenes, snRNA, snoRNA, misc RNA, and miRNAs (Figure 7 without *).

### Impact of Library Preparations for “Novel” lincRNA Detection

There was an average of 306 novel lincRNAs (251–369) detected in the PolyA samples with the minimum expression >100 reads; however, the novel lincRNAs for the NuGEN samples ranged from 724 to 1,822 (1,236) after adjusting for sequence depth at the similar expression level. The NuGEN protocol appeared more capable to detect totally novel lincRNAs. Collectively, there were 5,986 novel lincRNAs detected in these samples, of which 1752 (30%) were present in 2 or more samples with certain expression as mentioned above. Comparing with the Human lincRNA Catalog (Human Body Map lincRNAs, http://www.broadinstitute.org/genome_bio/human_lincrnas/?q=lincRNA_catalog) [18], we found 455 lincRNAs with 100% and 513 with over 50% coordinate overlap with the lincRNAs defined in the lincRNA Catalog. Of note, the lincRNAs in the Catalog were all identified from the RNA-seq data prepared by the PolyA protocol.

## Discussion

Library preparation protocol selection can be difficult when RNA source is limited, quality is compromised or targeted RNAs of interest may lack PolyA tails. RNA enrichment through the PolyA selection is the most common protocol for the RNA-seq experiment. The NuGEN protocol has been developed to overcome some of the limitations in the PolyA protocol. However, the in-depth analyses about the potential impacts on the downstream analyses from the two protocols have not been conducted, particularly for the applications in DEG detection in biological replicates, variant detection, and novel RNA discovery. In this study we compared the PolyA and the NuGEN preparation protocols with the same starting RNA with regard to these critical questions. Our results showed that the sequence data from the NuGEN library had lower alignment rates; a larger proportion of reads was aligned to noncoding regions (intronic or intergenic); fewer genes were captured; fewer DEGs between biological conditions were detected; the DEG overlap with the PolyA samples was low although the agreement for the common set was fairly good; a significantly higher number of SNVs was detected in the NuGEN sample than the PolyA sample, most of which were in intronic or intergenic regions.

The NuGEN protocol was initially developed to use a minute amount of RNA or deal with RNAs that are partially degraded. This technology has been applied to gene expression microarray [19], [20], [21] and adapted to RNA-seq recently [3], [6], [22]. The evaluation of the technology in microarray is largely limited to the gene expression correlation between the degraded and fresh material. As the applications of RNA-seq are far beyond gene expression and DEG detection, the impacts of different library preparations on result analysis and interpretation are important to apprehend. In the gene capture and quantification, our results are consistent with a previous report [6] in which the PolyA library detected more genes than the NuGEN preparation; a group of non-protein-coding transcripts had markedly higher read counts by the NuGEN protocol than the PolyA selection protocol. Unlike that study, our sequence depth for the NuGEN samples was higher with over 74% alignment rate (compared to 52%) yet we did not observe the NuGEN had an advantage to detect more known non-coding RNAs, particularly lincRNAs. In the comparison between the NuGEN Ovation® RNA-Seq system, the Illumina TruSeq™ poly-A enrichment and RiboMinus™ rRNA depletion, another study [22] also found the gene coverage in the NuGEN was much uneven than the PolyA or RiboMinus (higher average CV) and a larger proportion of reads was mapped to introns and intergenic regions (the PolyA was the lowest). The exon skipping and uneven coverage in the NuGEN samples may lead to potential false alternative splicing events.

The significant amount of reads mapped to intragenic and intergenic regions in the NuGEN samples have several implications. First, it may potentially uncover uncharacterized transcripts, non-coding or small RNAs. The observation that there are 4 times or more novel lincRNAs detected in the NuGEN samples than the PolyA protocol appears supporting the hypothesis. The little difference for the annotated lincRNAs as defined in the GENCODE may be the result of technology bias as most of RNA-seqs are performed through the PolyA selection. However, before we can validate these novel lincRNAs, this assertion can not be firmly established as the possibility of platform specific artifacts exists. Second, it contributes to a significantly higher number of SNVs. As most of the SNVs in these regions have no coverage from the PolyA samples, it is hard to assess their validity and the functional meaning of these SNVs needs to be further investigated.

When detecting DEGs in biological replicates, the NuGEN preparation tends to claim fewer DEGs than the PolyA preparation, which is mainly contributed by two possible reasons: 1) the NuGEN has fewer genes detected than the PolyA. 2) the gene expression in the NuGEN samples shows much higher variance which reduces statistical power to detect DEGs. Nevertheless, for the highly significant common genes detected in both protocols, a reasonable agreement is achieved and they are validated by RT-PCR. Therefore, for gene expression quantification and DEGs, the NuGEN protocol provides a good alternative when RNA material is limited although certain DEGs are missed. Increasing sample size with more biological replicates is needed to compensate the high variance of expression.

We were not able to assess the difference between the two protocols in fusion detection as these events mostly occur in tumors and no such events were detected in these cells in our analysis [23]. From what we observed in this study, it is conceivable that the data from the NuGEN protocol may be challenging as it has more exon skipping, lower number of reads mapped to exon-exon junction, and shorter fragment length.

In this study we used the data generated from two generations of Illumina sequencers (GAIIx for the PolyA and the HiSeq 2000 for the NuGEN) and the sequence outputs were different, which potentially confounded the results. To explore the possibility, we compared 4 tumor samples prepared by the PolyA protocol and sequenced by both the GAIIx and the HiSeq 2000. To make them more comparable, the sequence depths were standardized at ∼44 million. We found little difference between the data from the two sequencers in terms of alignment statistics, gene quantification and differently measured genes (Table S1, Figure S2 and S3). To evaluate the impact of sequence depths, we randomly drew about 40 million reads from each sample in our HMEC samples and then repeated the analyses. The results were very similar to the data when full data were used (Figure S4, S5, S6, S7). As the NuGEN samples had lower alignment rates and lower numbers of reads mapped to the RefSeq genes, the downsized samples had a lower power to detect DEGs between two conditions of biological replicates, which supports the observation that higher sequence depth was needed for the NuGEN samples (Note: the data without downsizing had closer numbers of reads mapped to genes in the NuGEN compared to the PolyA samples).

The main advantage of the NuGEN preparation is to use minute amount or partially degraded RNA such as from FFPE samples. We evaluated the potential issues for the former but were unable to conduct the comparisons for the latter as the PolyA would not work with the FFPE samples and the quality of RNA would complicate the comparisons. The study is intended to illustrate the biases from different protocols which need to be considered in data analysis and interpretation. In gene detection and DEGs, the NuGEN may miss a significant number of genes when sample size is small. SNVs, fusion genes or alternative splicing are more challenging and caution is needed for these results.

## Supporting Information

Figure S1 **Base quality score distribution for read 1 of the same sample sequenced with the PolyA and the NuGEN preparation.** All other samples and reads had the similar distribution. A: Reads from the PolyA sample; B: reads from the NuGEN sample.
(TIF)

Figure S2 **Gene expression correlation between GAIIx and HiSeq2000: A: raw gene count and log2 transformed. B: Normalized RPKM count and log2 transformed.**
(TIF)

Figure S3 **Unsupervised clustering and differentially measured genes between GAIIx and HiSeq200.** A: Unsupervised clustering for samples using all genes. The same samples sequenced by GAIIx and HiSeq2000 clustered tightly. B: P value distribution for differentially measured genes. Only very few genes were differentially measured between the two platforms, much fewer than random noises.
(TIF)

Figure S4 **Alignment and gene capture statistics for the PolyA and NuGEN after standardizing sequencing depth at ∼40 million for all samples.**
(TIF)

Figure S5 **Gene expression correlation between the PolyA and the NuGEN after sequence depth normalization (4 sample pairs shown).** A: raw gene count without any normalization. B: RPKM normalized data.
(TIF)

Figure S6 **Common DEGs detected between the PolyA and the NuGEN preparations after standardizing read depths to 40 million.**
(TIF)

Figure S7 **Number of SNVs detected in each sample by two library preparations.** Much more “SNVs” are in the NuGEN sample than the PolyA samples.
(TIF)

Table S1 **Alignment statistics for 4 samples sequenced by both GAIIx and HiSeq2000.** The read depths were standardized to the similar number by randomly drawing reads from the samples with higher depths. The same samples have the very similar alignment statistics.
(DOCX)

Table S2 **Command and parameters for Cufflink and Scripture used for novel lincRNA identification**.
(DOCX)
