# Supplementary figures and images for: Impact of Library Preparation on Downstream Analysis and Interpretation of RNA-Seq Data: Comparison between Illumina PolyA and NuGEN Ovation Protocol
Source: PLoS One. 2013 Aug 19;8(8):e71745. doi: 10.1371/journal.pone.0071745 (PMC3747248; doi:10.1371/journal.pone.0071745)

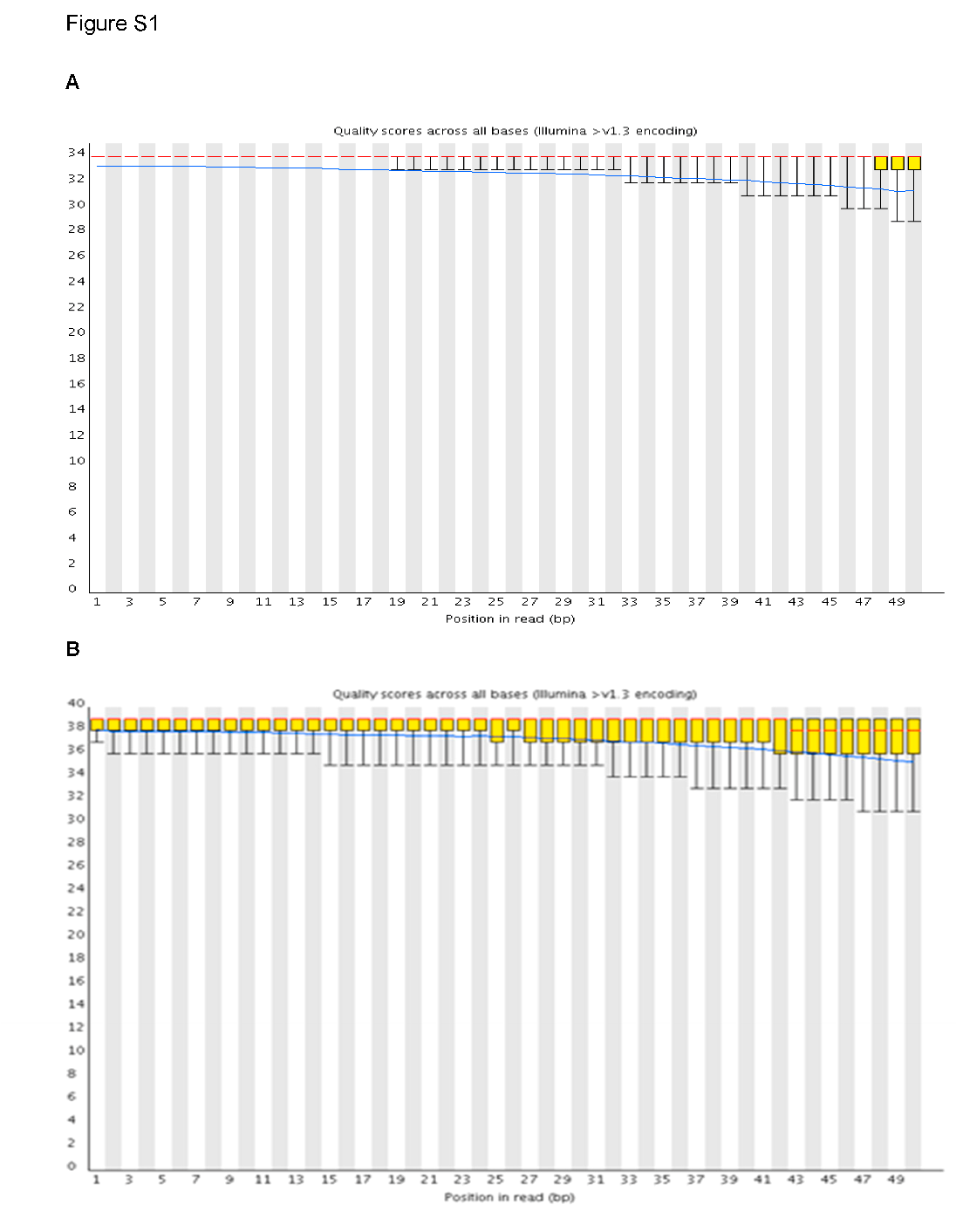

Supplement: Figure S1 — Base quality score distribution for read 1 of the same sample sequenced with the PolyA and the NuGEN preparation. All other samples and reads had the similar distribution. A: Reads from the PolyA sample; B: reads from the NuGEN sample. (TIF) [file pone.0071745.s001.tif]

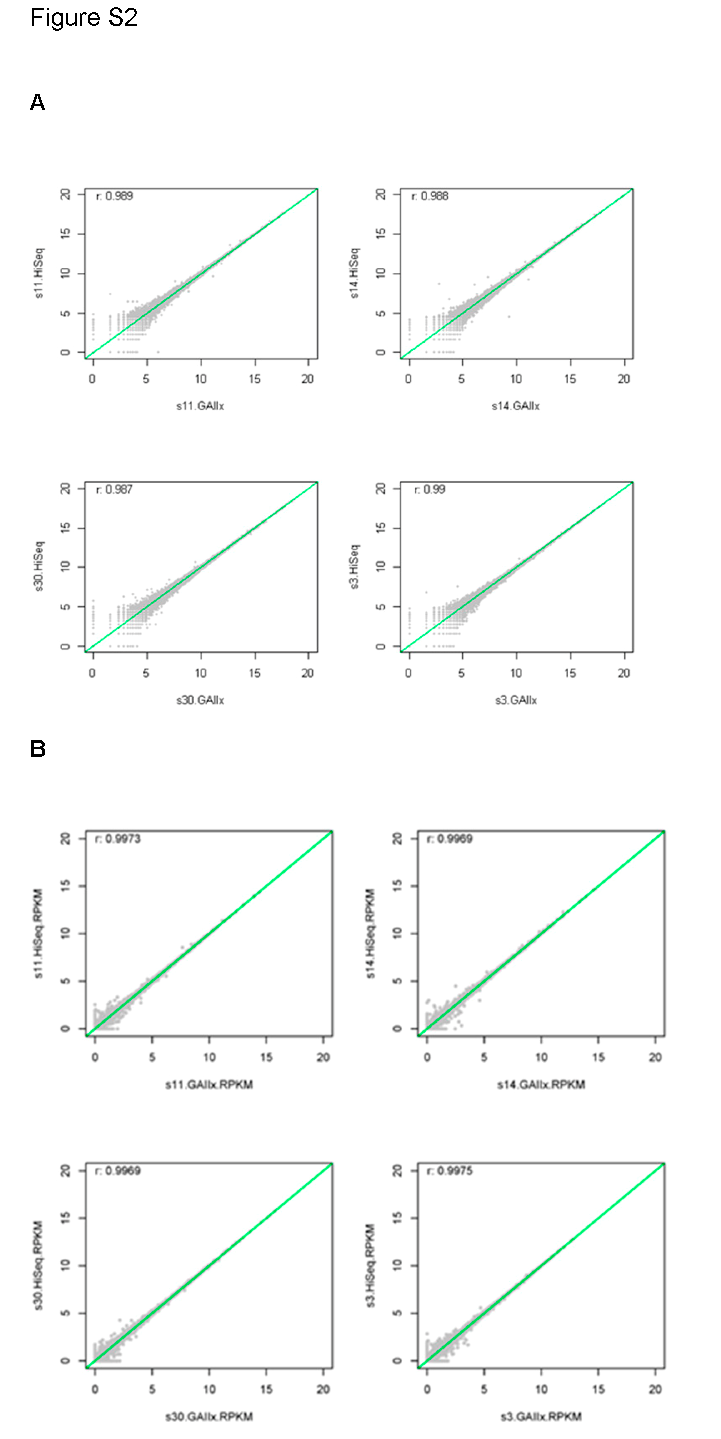

Supplement: Figure S2 — Gene expression correlation between GAIIx and HiSeq2000: A: raw gene count and log2 transformed. B: Normalized RPKM count and log2 transformed. (TIF) [file pone.0071745.s002.tif]

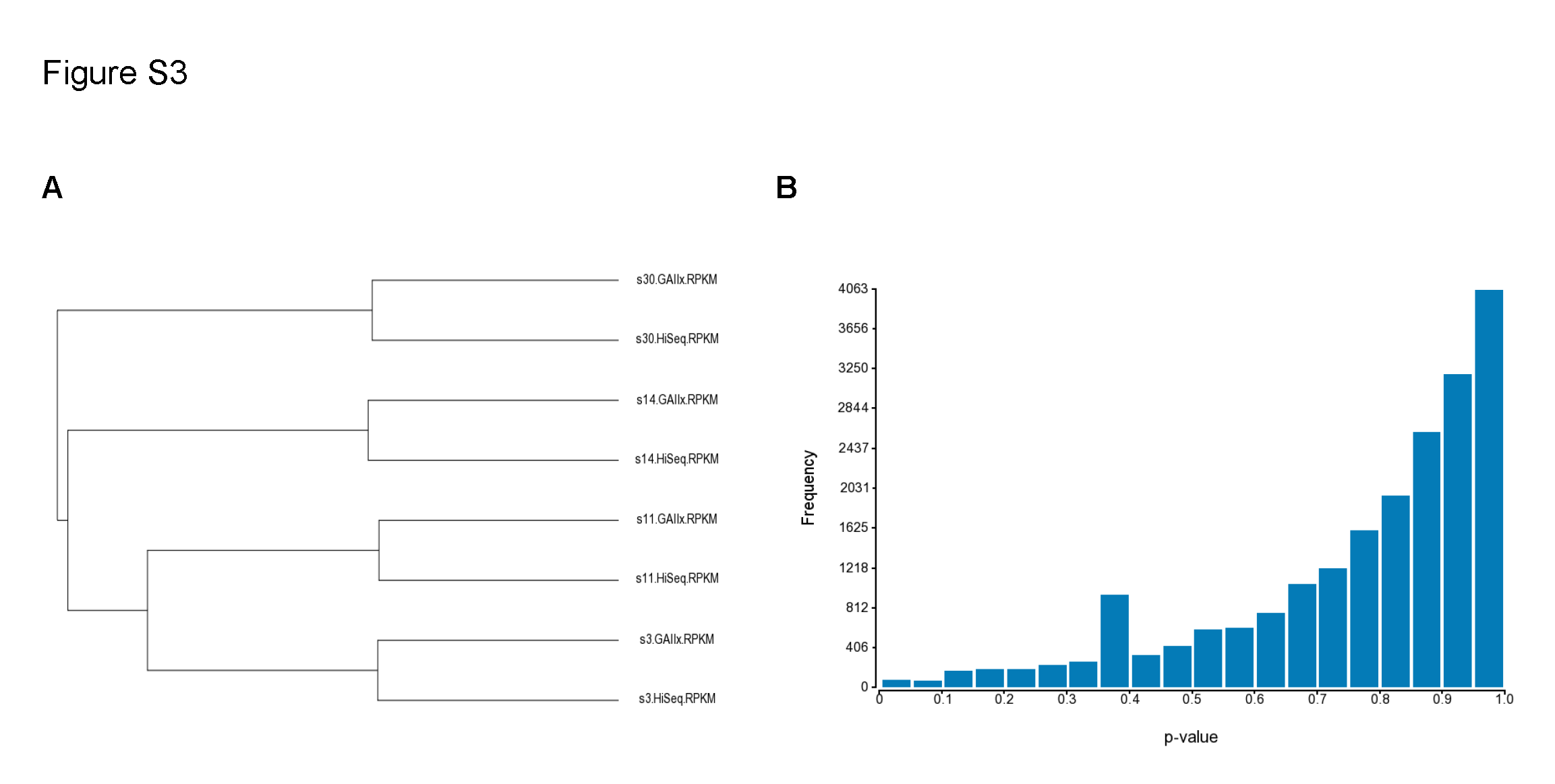

Supplement: Figure S3 — Unsupervised clustering and differentially measured genes between GAIIx and HiSeq200. A: Unsupervised clustering for samples using all genes. The same samples sequenced by GAIIx and HiSeq2000 clustered tightly. B: P value distribution for differentially measured genes. Only very few genes were differentially measured between the two platforms, much fewer than random noises. (TIF) [file pone.0071745.s003.tif]

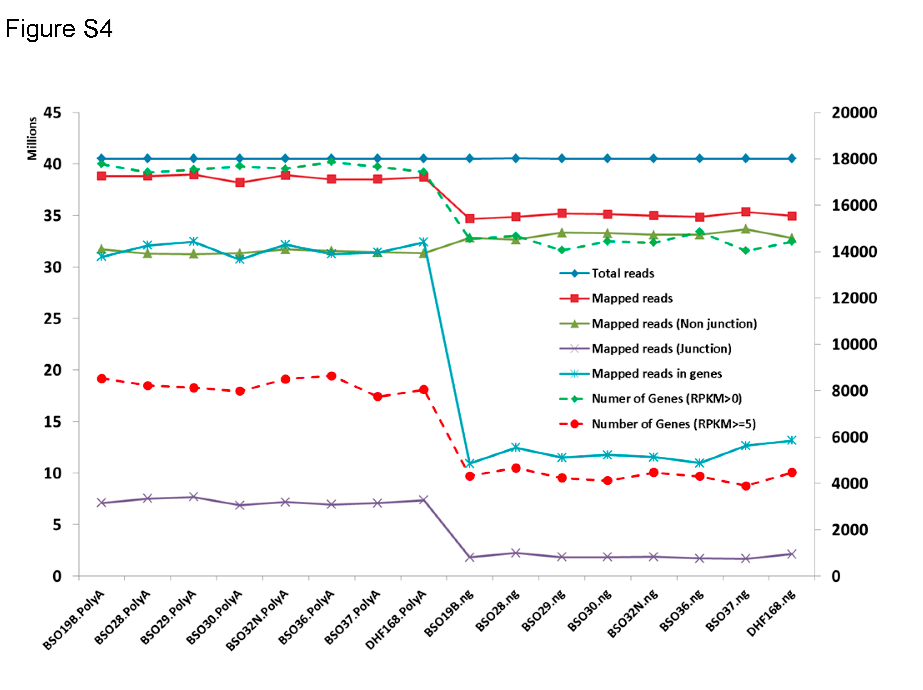

Supplement: Figure S4 — Alignment and gene capture statistics for the PolyA and NuGEN after standardizing sequencing depth at ∼40 million for all samples. (TIF) [file pone.0071745.s004.tif]

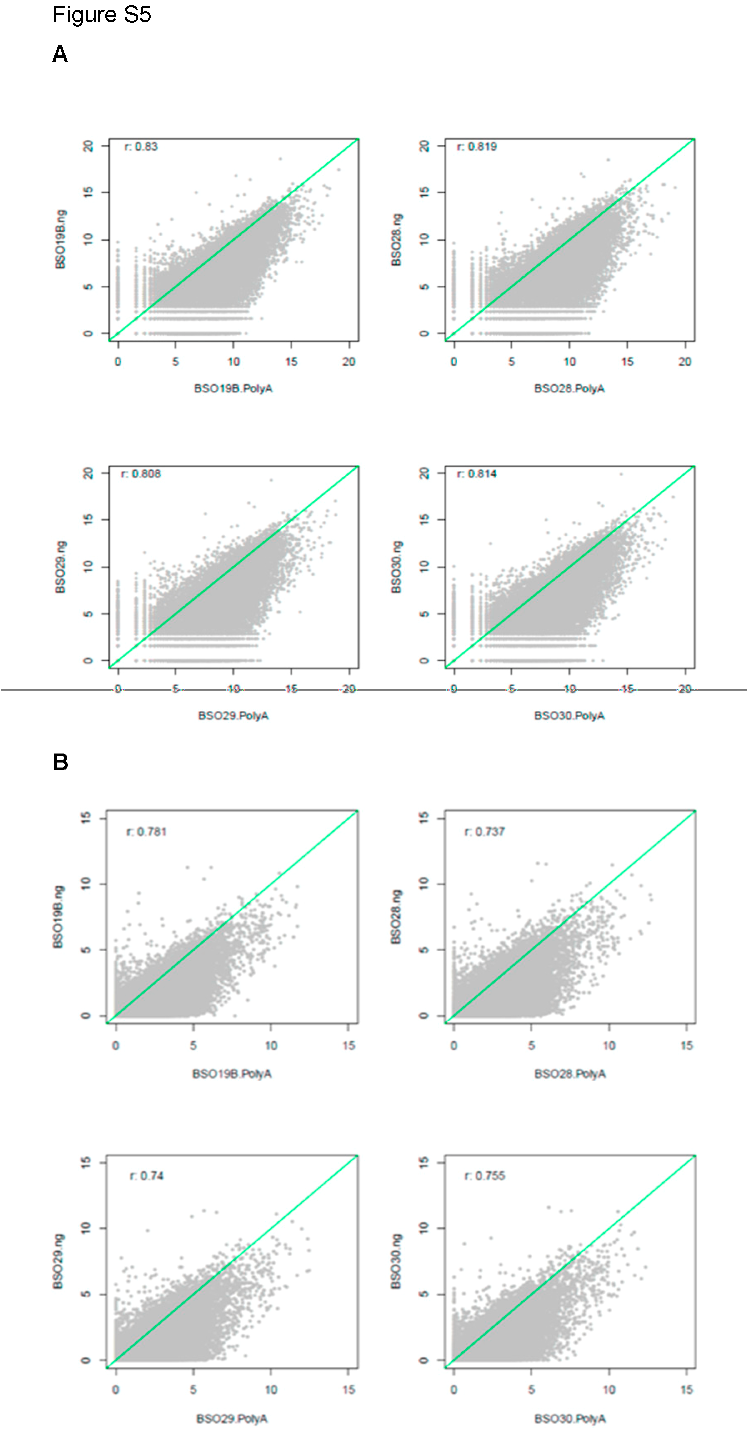

Supplement: Figure S5 — Gene expression correlation between the PolyA and the NuGEN after sequence depth normalization (4 sample pairs shown). A: raw gene count without any normalization. B: RPKM normalized data. (TIF) [file pone.0071745.s005.tif]

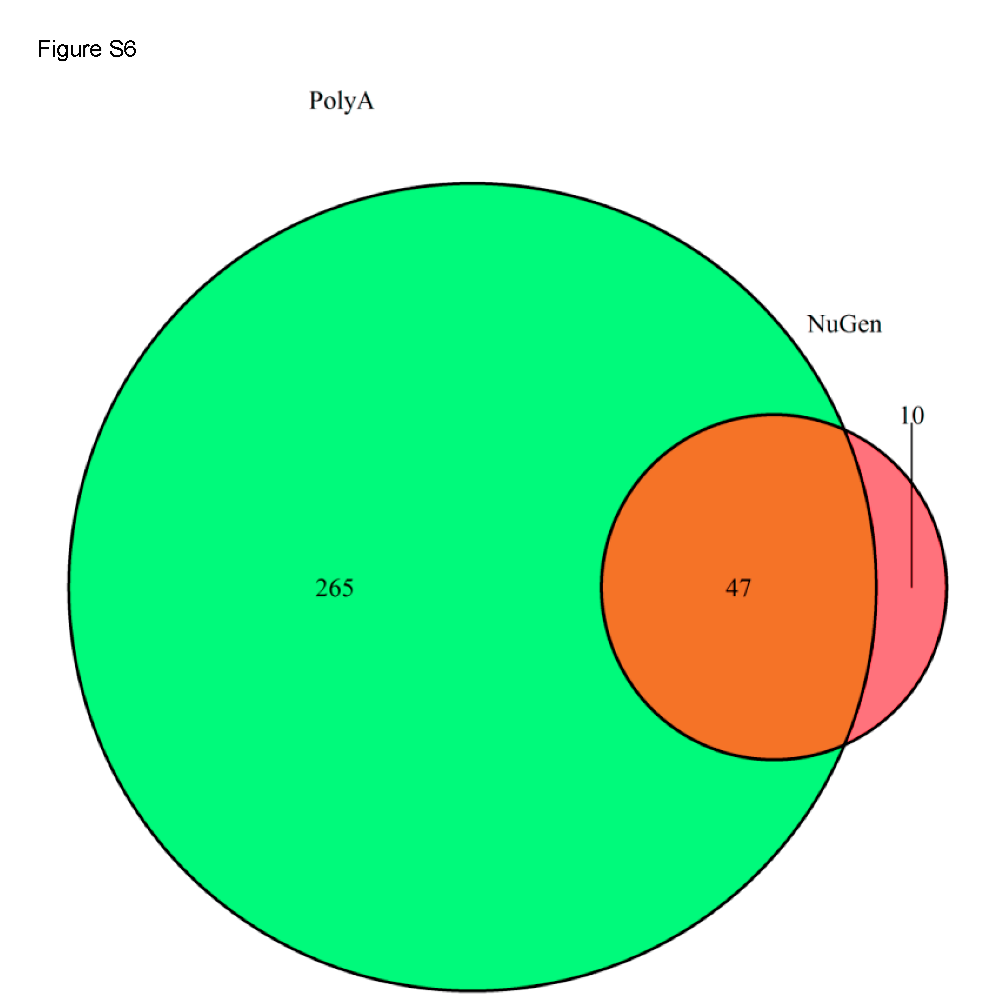

Supplement: Figure S6 — Common DEGs detected between the PolyA and the NuGEN preparations after standardizing read depths to 40 million. (TIF) [file pone.0071745.s006.tif]

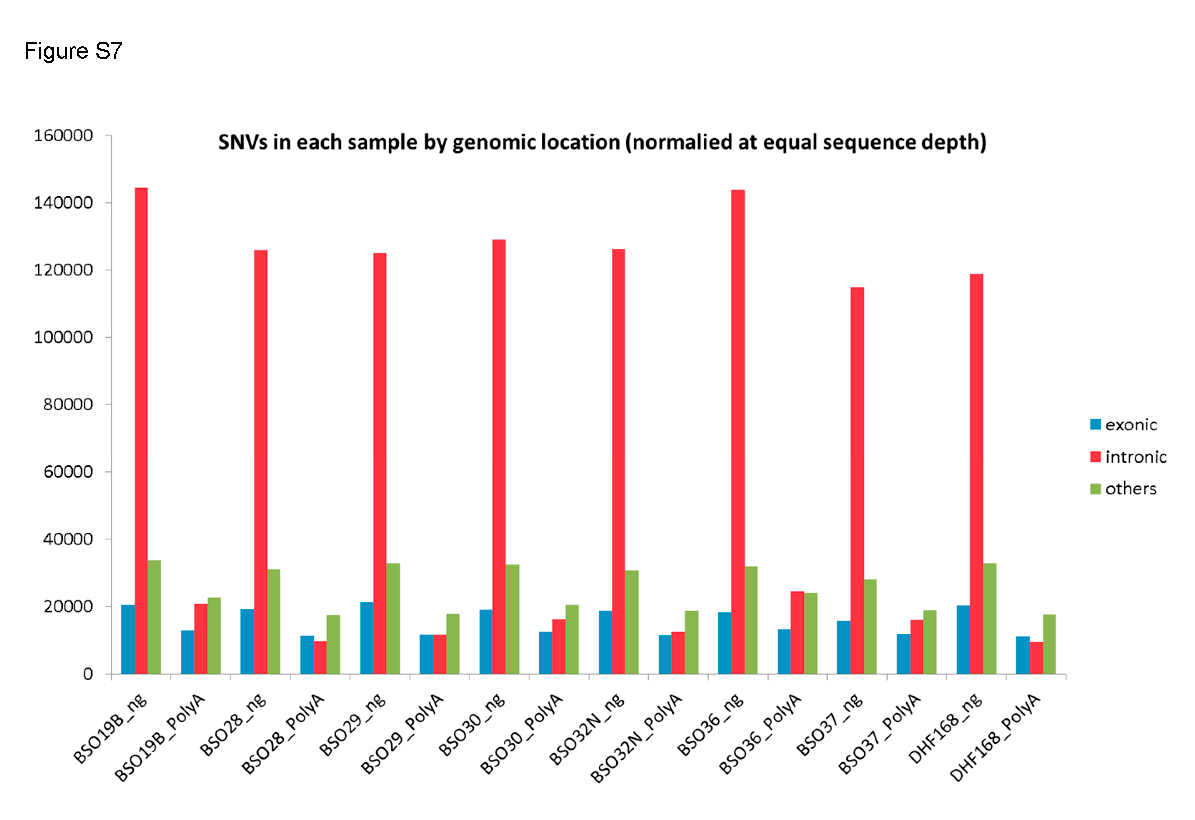

Supplement: Figure S7 — Number of SNVs detected in each sample by two library preparations. Much more “SNVs” are in the NuGEN sample than the PolyA samples. (TIF) [file pone.0071745.s007.tif]
