# Supplementary material for: Impact of Library Preparation on Downstream Analysis and Interpretation of RNA-Seq Data: Comparison between Illumina PolyA and NuGEN Ovation Protocol
Source: PLoS One. 2013 Aug 19;8(8):e71745. doi: 10.1371/journal.pone.0071745 (PMC3747248; doi:10.1371/journal.pone.0071745)
Supplement: Table S1 — Alignment statistics for 4 samples sequenced by both GAIIx and HiSeq2000. The read depths were standardized to the similar number by randomly drawing reads from the samples with higher depths. The same samples have the very similar alignment statistics. (DOCX) [file pone.0071745.s008.docx]

| Sample. Sequencer | Total reads | Mapped | %Mapping | Genome reads | %Genome | Junction reads | %Junction reads |
| --- | --- | --- | --- | --- | --- | --- | --- |
| s11.GAIIx | 44,122,160 | 42,333,458 | 95.9 | 33,683,911 | 76.3 | 8,649,547 | 19.6 |
| s11.HiSeq | 44,754,656 | 42,328,390 | 94.6 | 33,673,647 | 75.2 | 8,654,743 | 19.3 |
| s14.GAIIx | 44,124,570 | 42,353,623 | 96 | 33,027,360 | 74.9 | 9,326,263 | 21.1 |
| s14.HiSeq | 43,846,418 | 41,495,892 | 94.6 | 32,379,169 | 73.8 | 9,116,723 | 20.8 |
| s30.GAIIx | 44,510,720 | 42,578,818 | 95.7 | 32,920,238 | 74 | 9,658,580 | 21.7 |
| s30.HiSeq | 44,127,584 | 41,661,712 | 94.4 | 32,173,796 | 72.9 | 9,487,916 | 21.5 |
| s3.GAIIx | 44,267,842 | 42,386,537 | 95.8 | 34,433,909 | 77.8 | 7,952,628 | 18 |
| s3.HiSeq | 43,944,644 | 41,652,227 | 94.8 | 33,790,430 | 76.9 | 7,861,797 | 17.9 |
