# Supplementary material for: Impact of Library Preparation on Downstream Analysis and Interpretation of RNA-Seq Data: Comparison between Illumina PolyA and NuGEN Ovation Protocol
Source: PLoS One. 2013 Aug 19;8(8):e71745. doi: 10.1371/journal.pone.0071745 (PMC3747248; doi:10.1371/journal.pone.0071745)
Supplement: Table S2 — Command and parameters for Cufflink and Scripture used for novel lincRNA identification. (DOCX) [file pone.0071745.s009.docx]

| Cufflinks: |
| --- |
| cufflinks -o [alignment directory] -G [gencode GTF] -p 8 -g [gencode GTF] --multi-read-correct --upper-quartile-norm [BAM file] |
| Scripture: |
| java -Xmx2000m -jar scripture-beta2.jar -alignment [BAM file] -maskFileDir [Any nucleotide sequence to be masked] -out [output BED file] -chr [chr number, e.g., chr6] -sizeFile [file containing chromosome numbers and their sizes, similar to allchr.fa.fai] -chrSequence [FASTA sequence of respective chr] |
